# Lipopolysaccharides induce a RAGE-mediated sensitization of sensory neurons and fluid hypersecretion in the upper airways

**DOI:** 10.1038/s41598-021-86069-6

**Published:** 2021-04-16

**Authors:** Manoj Nair, Santosh Jagadeeshan, George Katselis, Xiaojie Luan, Zeinab Momeni, Nicolas Henao-Romero, Paulos Chumala, Julian S. Tam, Yasuhiko Yamamoto, Juan P. Ianowski, Verónica A. Campanucci

**Affiliations:** 1 Department of Anatomy, Physiology and Pharmacology (APP), College of Medicine, University of Saskatchewan, 107 Wiggins Road, Saskatoon, SK S7N 5E5 Canada; 2 Department of Medicine, College of Medicine, University of Saskatchewan, 107 Wiggins Road, Saskatoon, SK S7N 5E5 Canada; 3 Department of Biochemistry and Molecular Vascular Biology, Kanazawa University Graduate School of Medical Science, Kanazawa, 920-8640 Japan; 4 Department of Medicine, Division of Respirology, College of Medicine, University of Saskatchewan, 107 Wiggins Road, Saskatoon, SK S7N 5E5 Canada

**Keywords:** Neuroscience, Physiology, Diseases

## Abstract

Thoracic dorsal root ganglia (tDRG) contribute to fluid secretion in the upper airways. Inflammation potentiates DRG responses, but the mechanisms remain under investigation. The receptor for advanced glycation end-products (RAGE) underlies potentiation of DRG responses in pain pathologies; however, its role in other sensory modalities is less understood. We hypothesize that RAGE contributes to electrophysiological and biochemical changes in tDRGs during inflammation. We used tDRGs and tracheas from wild types (WT), RAGE knock-out (RAGE-KO), and with the RAGE antagonist FPS-ZM1, and exposed them to lipopolysaccharides (LPS). We studied: capsaicin (CAP)-evoked currents and action potentials (AP), tracheal submucosal gland secretion, RAGE expression and downstream pathways. In WT neurons, LPS increased CAP-evoked currents and AP generation, and it caused submucosal gland hypersecretion in tracheas from WT mice exposed to LPS. In contrast, LPS had no effect on tDRG excitability or gland secretion in RAGE-KO mice or mice treated with FPS-ZM1. LPS upregulated full-length RAGE (encoded by Tv1-RAGE) and downregulated a soluble (sRAGE) splice variant (encoded by *Mmus*RAGEv4) in tDRG neurons. These data suggest that sensitization of tDRG neurons contributes to hypersecretion in the upper airways during inflammation. And at least two RAGE variants may be involved in these effects of LPS.

## Introduction

Sensitization of peripheral neurons is part of the neurogenic abnormalities that contribute to chronic sensory pathologies. The mechanisms involved in heightened sensory responses have been studied mostly in the context of neuropathic pain; however, they can also play critical roles in other sensory modalities, such as those that trigger reflexes in the airways. The neurogenic control of the airways is mediated by the combined action of sensory and autonomic fibers and the airway intrinsic plexuses, resulting in the regulation of airway function, and the maintenance of airway homeostasis by compensatory reflexes^1–3^. One of the well-studied mechanisms protecting the airways is the production of airway surface liquid by submucosal glands that line the upper airways^2^. The secretion from submucosal glands is predominantly under parasympathetic control. The sensory inputs arise from vagal sensory (afferent) neurons innervating the upper airways, with cell bodies in the jugular/nodose ganglia^2,4,5^. In addition to the vagal/parasympathetic control, sensory information is also processed by neurons whose cell bodies are located in thoracic (T1-T6) dorsal root ganglia (tDRG)^6,7^, which activate sympathetic efferent fibers also found in the vicinity of the submucosal glands^2^. Other than their different anatomical locations, both DRG and jugular/nodose sensory neurons express typical sensory markers such as the transient receptor potential vanilloid 1 (TRPV1), substance P (SP), calcitonin gene-related peptide (CGRP) and lectin IB4^7–9^. It is generally accepted that the role of tDRG neurons is less important than their vagal counterparts since most of the known airway reflexes can be significantly suppressed by bilateral vagotomy^2^. Nevertheless, the synergistic effect of multiple neurotransmitters released on the airways by peripheral innervation, together with the possibility of direct gland stimulation by sensory axon reflexes, or indirectly via central reflexes^1,2^, could all converge during inflammation, playing a significant role in the regulation of the airways under pathological conditions.

Sensitization of DRG neurons contribute to chronic inflammatory conditions of the airways, such as asthma and chronic obstructive pulmonary disease (COPD)^10,11^. One of the known insults that can affect normal sensory neuron function is exposure to pathogens invading the airways. Bacterial lipopolysaccharides (LPS) are known for their actions on the toll-like receptor 4 (TLR4), a member of the pattern-recognition receptor (PRR) family^12,13^, which leads to potentiation of TRPV1 currents and subsequent increase of intracellular Ca^2+^ concentration. In addition, the interaction of LPS with TLR4 leads to activation of signaling cascades such as the nuclear factor kappa B (NFkB) and mitogen-activated protein kinases (MAPKs)^13^, which in turn induce the release of pro-inflammatory cytokines. However, in addition to stimulating TLR4, LPS also binds to the receptor for advanced glycation end-products (RAGE)^14^. RAGE, a member of the immunoglobulin protein family^15,16^, is a multi-ligand receptor that is known to interact with advanced glycation end-products (AGEs), certain members of the S100/calgranulin family and pro-inflammatory proteins such as high-mobility group box 1 (HMGB1) and β-integrin Mac-1^17–19^. The interaction between RAGE and many of its ligands induces oxidative stress, probably through activation of NADPH oxidase^20–24^, and the MAPK pathway^25–30^.

In humans, increased RAGE signalling has been linked to chronic diseases of the airways, such as chronic COPD, cystic fibrosis (CF), and asthma^31–33^. However, little is known about how RAGE contributes to either physiology or pathology in the airways^34^.

Alternative splicing studies have identified a remarkable diversity of RAGE transcriptional and translational variants, many of which are expressed as tissue-specific isoforms^35,36^ that are yet to be identified in airway peripheral neurons. Therefore, identifying RAGE isoforms specific to airway peripheral neurons under pathological conditions may help better understand inflammatory processes that affect the function of the airways.

In this study, we hypothesize that LPS induces electrophysiological changes in tDRG neurons consistent with neuronal sensitization, which leads to airway submucosal gland hypersecretion. We further propose that these changes require the expression and/or regulation of specific RAGE variants. Our findings revealed that in response to LPS treatment, full-length membrane-associated RAGE expression, encoded by Tv1-RAGE, is upregulated and required for the sensitization of tDRG neurons. The latter was accompanied by the downregulation of a soluble splice variant encoded by *Mmus*RAGEv4. Our findings correlate with the effect of LPS treatment in live mice, which triggers submucosal gland hypersecretion in the trachea, an effect that required neuronal activity. This response is absent in RAGE knock-out (RAGE KO) mice or wild type animals in which RAGE function has been inhibited by FPS-ZM1, indicating that the effect of LPS was dependent on RAGE expression and function. Therefore, our study suggests that RAGE-mediated changes in tDRG neurons may contribute to airway submucosal gland hypersecretion in the presence of bacterial infection.

## Material and methods

### Mice

A colony of RAGE KO mice on a C57BL/6 background was maintained by breeding heterozygous mice, as previously described^24^. Heterozygous mice were generated by back-crossing RAGE KO (homozygous) mice^37^ with C57BL/6 wild type (WT) mice. All experiments used thoracic (T1–T4) dorsal root ganglia (tDRG) from homozygous (RAGE KO) mice and their C57BL/6 (wild type) littermates. Mice were genotyped using polymerase chain reaction as previously described^24^. Some WT mice were injected with either the RAGE antagonist FPS-ZM1 (Calbiochem, Sigma) (3 mg/kg/day, i.p.^38^) or a corresponding volume of saline (daily, i.p.) for 48 h before starting experiments. All in vitro experiments involving neuronal primary cultures were done with neonatal pups (P0–P5).

This work was approved by the University of Saskatchewan Animal Research Ethics Board (Campanucci: protocols 20090082 and 20150051) and adhered to the Canadian Council on Animal Care guidelines for humane animal use.

### Primary tDRG cultures

Bilateral thoracic (T1–T4) DRG neurons were harvested and cultured from neonatal (P0–P5) mice as previously described^39^. Briefly, ganglia were removed under sterile conditions and enzymatically dissociated at 37 °C in Hank’s balanced salt solution (HBSS) containing trypsin (180–200 U/ml; Worthington, Freehold, NJ, USA) and buffered with HEPES (pH 7.4). The resulting cell suspension was washed twice in serum-containing Leibovitz's L-15 medium to inactivate the trypsin and plated on laminin-coated glass-bottom Petri dishes (35 mm) made in-house. The neurons were grown in L-15 medium supplemented with vitamins, cofactors, penicillin–streptomycin, 5 mM glucose, 5% rat serum and NGF (10 ng/ml; Alomone Labs, Jerusalem, Israel). Cultures were maintained at 37 °C in a humidified atmosphere of 95% air-5% CO_2_ and fed every 4 days with growth media. To eliminate non-neuronal cells, cultures were treated with cytosine arabinoside (10 μM; Sigma, St. Louis, MO, USA) from days 2 to 4, which prevents the proliferation of non-neuronal cells resulting in a neuron-enriched primary culture. Established DRG cultures were incubated for 24 h at 37 °C; (5% CO_2_; humidified) in growth media alone (control) or with 1 µg/ml lipopolysaccharide (LPS, Sigma-Aldrich, MO, USA)-supplemented growth media. To inhibit RAGE function, some cultures were incubated with either the RAGE antagonist FPS-ZM1 (10 μM), or the vehicle (0.01% dymethyl sulfoxide), for 48 hr before electrophysiological recording.

### Electrophysiology

Medium-sized DRG neurons (~ 25–35 µm) were selected for whole-cell recording^40^. Membrane currents were recorded with an Axopatch 200B amplifier (Molecular Devices, Palo Alto, CA) equipped with a 1 GΩ cooled head-stage feedback resistor and a Digidata 1400A analog-to-digital converter (Molecular Devices), and stored on a personal computer. Current- and voltage-clamp protocols, data acquisition, and analysis were performed using pClamp 10 (Molecular Devices) and Origin 9.0 software package (OriginLab Corporation, Northampton, MA, USA). Patch pipettes were made using thin-wall borosilicate glass capillaries (World Precision Instruments, FL, USA) using a vertical puller (PC 10; Narishige Scientific Instrument Lab., Tokyo, Japan) and polished with a microforge (Narishige) to a final resistance of 3–8 MΩ when filled with intracellular recording solution. In most experiments, 75% of the series resistance was compensated, and junction potentials were canceled at the beginning of the experiment. Recording electrodes were filled with the following intracellular solution (in mM): 65 KF, 55 KAc, 5 NaCl, 0.2 CaCl_2_, 1 MgCl_2_, 10 EGTA, 2 MgATP, and 10 HEPES, and pH was adjusted to 7.2 with KOH (all from Sigma-Aldrich). Cultured neurons were perfused continuously at 1 ml/min with control perfusion solution consisting of (in mM): 140 NaCl, 5.4 KCl, 0.33 NaH_2_PO_4_, 0.44 KH_2_PO_4_, 2.8 CaCl_2_, 0.18 MgCl_2_, 10 HEPES, 5.6 glucose, 2 glutamine, 0.001 atropine and 5 μg/ml phenol red; pH was adjusted to 7.4 with NaOH (all from Sigma-Aldrich). Once neurons were in the whole-cell patch-clamp configuration, we allowed the cell to stabilize for 5 min before collecting data. Action potentials were generated in current-clamp mode by injection of a series of depolarizing current steps at 100 pA increments for 500 ms. All other experiments were carried out under voltage-clamp mode. A fast-step perfusion system was used to deliver either control or capsaicin (CAP; 1–10 µM; Sigma-Aldrich)-containing extracellular solution at 1 ml/min perfusion rate.

### Intranasal LPS application, trachea preparation, and secretion assay

Adult (2 to 4-month-old) female and male WT, RAGE-KO, WT injected with FPS-ZM1, and WT injected with saline mice were treated intranasally with LPS (40 µg/kg body weight), or with equivalent volume of PBS as controls (volume was 6 μl per nostril). After 6 h^41,42^, the animals were euthanized, and the tracheas were dissected as previously described^43,44^. Briefly, tracheas were dissected and placed in ice-cold Krebs–Ringer bicarbonate buffer (pH = 7.4) equilibrated with 95% O_2_–5% CO_2_ and used for experimentation within 60 min for secretion assays.

We used a modification of a secretion assay developed by Quinton^45^ as explained elsewhere^43,44,46,47^. Briefly, the trachea was cut dorsally along its length and placed in a custom-built chamber mucosal side up so that the serosal side was bathed in ∼ 60 μl Krebs solution, and the mucosal side was exposed to air. The luminal, mucosal surface was gently cleaned with absorbent paper, dried with a stream of air, and coated with ∼ 5 μl of mineral oil (water-saturated) between the first and third cartilage rings. The preparation was then placed in a temperature-controlled chamber (TC-324B; Warner Instruments, Hamden, CT) maintained at 37 °C and equilibrated with warmed, humidified 95% O_2_–5% CO_2_. Lidocaine (Sigma-Aldrich) and tetrodotoxin (TTX, Alomone Labs) were diluted to final concentrations with warmed bath solution equilibrated with 95% O_2_–5% CO_2_ and were added to the serosal side by complete bath replacement. Capsaicin (CAP) was dissolved in mineral oil and added to the luminal side of the preparation.

Fluid secretion by individual glands formed droplets under oil. Images of the droplets formed in the lumen of the trachea were taken every 30 s using a digital camera (MiniVid; LW Scientific, Lawrenceville, GA) and stored for offline analysis. Stored images were analyzed using ImageJ 1.32 J (National Institutes of Health; NIH). Secretion volumes were calculated, as previously described, by assuming the mucus droplet to be spherical using the formula volume (V) = 4/3πr^3^, where r is the radius^43^. The following inclusion criteria for individual droplets were used: (1) circular outline so that a spherical shape could be assumed. (2) Clear edges to allow accurate measurement of the radius. And (3) no fusion with neighboring droplets. The viability of the preparation was tested at the end of each experiment by measuring the response to the cholinergic agonist, carbachol (Sigma-Aldrich)^43^. Glands that failed to respond to carbachol (< 5%) were excluded from the analysis. The secretion rate was calculated by fitting the volume vs. time plots using linear regression, and the slopes were taken as the secretion rates using at least four points. The r^2^ value for such linear fits was > 0.8.

### Western blotting

We used whole extracts from whole tDRGs collected from adult WT mice for Western blotting^48^. Whole tDRG extracts were homogenized in ice-cold CelLytic MT Cell Lysis Reagent (Sigma-Aldrich) containing a protease and phosphatase inhibitor cocktail. We used 4 replicas per condition (PBS-control and LPS), and each replica was generated from 2 adult mice. Equal amounts of protein were loaded per group, separated on 12% SDS polyacrylamide gels and then electrotransferred onto a PVDF or nitrocellulose membrane (Bio-Rad Laboratories, Hercules, CA, USA). Membranes were incubated overnight at 4 °C with the following primary antibodies: rabbit anti-RAGE (1:1000; Abcam) and mouse anti-β-actin (1:2000; Sigma); followed by horseradish peroxidase-conjugated goat anti-rabbit or goat anti-mouse secondary antibodies (1:20,000; Bio-Rad Laboratories). Protein signals were visualized using enhanced chemiluminescence reagents (Bio-Rad) and quantified by densitometry using ImageJ software (NIH, Bethesda, MD, USA).

### Mass spectrometry-based proteomic analysis

A more detailed version of the methods is available as “Supplementary data”. Primary WT mouse thoracic (T1–T4) DRG suspensions were prepared as described above and plated onto specialized cell culture dishes (Sarstedt, Nümbrecht, Germany). Membrane and soluble fractions from approximately 80,000 cells per experimental group (control or LPS; 4 separate platings) were obtained using the ProteoExtract Native Membrane Protein Extraction Kit (EMD Millipore, MA, USA) and processed as per the manufacturer’s instructions and stored at − 80 °C. Protein concentrations were determined by NanoDrop BioTek ELx808 (BioTek Instruments Inc., VT, USA). Protein samples were reduced, alkylated, and digested with trypsin in-solution following a modified version of a previously published protocol^49^. Tryptic peptides were first subjected to strong cation exchange (SCX) fractionation using a SCX SpinTips sample preparation kit (Protea Biosciences, Morgantown, WV, USA), according to manufacturer’s instructions, before they were analyzed by liquid chromatography-tandem mass spectrometry (LC–MS/MS). MS analysis was performed using an Agilent 6550 iFunnel quadrupole time-of-flight (QTOF) mass spectrometer equipped with an Agilent 1260 series LC instrument and an Agilent Chip Cube LC–MS interface (Agilent Technologies, Mississauga ON, Canada). Chromatographic separation of peptides was accomplished using a high-capacity Agilent HPLC Polaris Chip and a linear gradient solvent system consisting of formic acid, water, and acetonitrile. Positive-ion electrospray tandem mass spectral data were collected over a mass range of 100–1700 mass/charge.

Tandem mass spectra were extracted from raw data and processed against the mouse NCBI non-redundant database and a custom database (containing all known mouse RAGE protein isoforms) using Spectrum Mill (Agilent Technologies Canada Ltd., Mississauga, ON, CA). Search parameters included a fragment mass error of 50 parts per million (ppm), a parent mass error of 20 ppm, trypsin cleavage specificity, and carbamidomethyl as a fixed modification of cysteine. Variable modifications included: carbamylated and acetyl-lysine, oxidized methionine, pyroglutamic acid, deamidated asparagine, and phosphorylated serine, threonine, and tyrosine. Spectra were also searched against semi-trypsin non-specific C- and N-termini. Spectrum Mill results were validated at peptide and protein levels (1% false discovery rate, FDR), and spectral counts and intensities were used to report relative quantification of proteins. Mass Profiler Professional (MPP, version 15.0, Agilent, Santa Clara, CA, USA) software was used for statistical analysis using one-way ANOVA. A cut-off value of p < 0.05 and the Benjamini and Hochberg FDR set at < 1% were used to obtain statistically significant results. In addition, a fold change (FC) of ≥ 2 and < 0.5 in spectral intensities with respect to control were considered to classify proteins as up- and down-regulated, respectively.

### Statistical analysis

To compare mean ± SEM values of EC_50_ from WT and RAGE KO dose–response curves, we used unpaired *t* test with Welch correction. For electrophysiology (peak amplitude, current density, ionic charge, and action potentials) and secretion assays, mean ± SEM values were compared between treatments (LPS or PBS) and genotype, RAGE antagonist or neural blockers (TTX and lidocaine) by 2-way ANOVA followed by Tukey's multiple comparisons test.

## Results

### WT and RAGE KO tDRG neurons sensitivity to CAP

**Figure 1:**
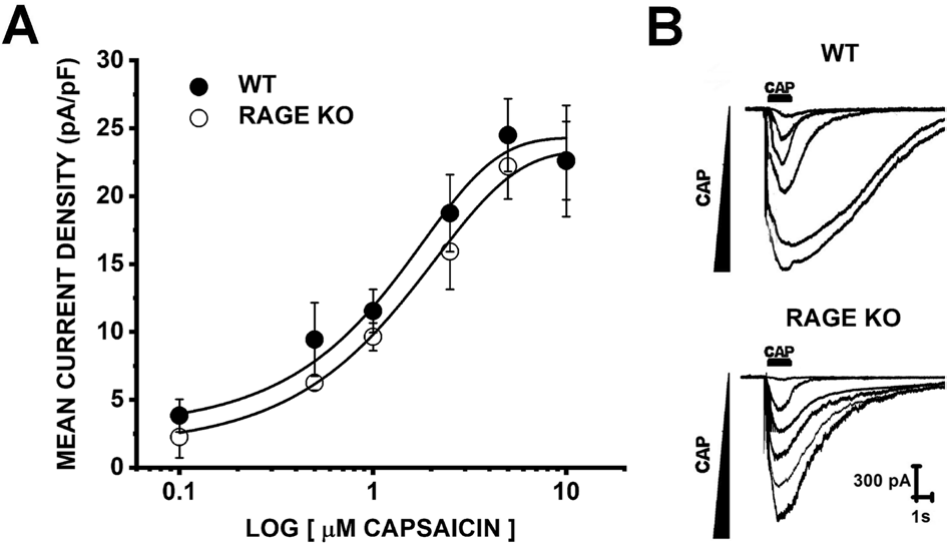
Dose–response of CAP-evoked currents in wild-type and RAGE KO tDRG neurons. Concentration dependency of CAP-evoked currents from cultured tDRG neurons from wild-type (WT) and RAGE-knockout (RAGE KO) mice by whole-cell patch clamp electrophysiology. DRGs neurons were clamped at -60 mV and exposed to CAP for 1 s. Data points indicate mean current density (pA/pF) at 0.1 (WT n = 10; RAGE KO n = 10), 0.5 (WT n = 7; RAGE KO n = 8), 1 (WT n = 16; RAGE KO n = 16), 2.5 (WT n = 6; RAGE KO n = 7), 5 (WT n = 9; RAGE KO n = 8) and 10 µM CAP (WT n = 5; RAGE KO n = 9). The data were fitted with a logistic Hill function—WT r^2^ = 0.98079; RAGE KO r^2^ = 0.99648. Error bars indicate mean ± SEM. The EC_50_ of CAP in wild-type and RAGE KO was 1.50 ± 1.29 µM and 2.01 ± 1.04 µM, respectively. Both EC_50_ values were not significantly different according to unpaired *t*-test (Welch correction).

To determine a submaximal working concentration of capsaicin (CAP) that would allow us to observe potential increases and decreases in whole-cell currents, we generated a dose–response relationship to increasing concentrations of CAP in tDRG cultured neurons from WT and RAGE KO mice (Fig. 1). Cultured neurons were briefly exposed to CAP (0.1, 0.5, 1, 2.5, 5 and 10 µM; 1 s; n = 6–16 per concentration) and the data were fitted with a logistic Hill function (r^2^ = 0.98079 for WT, and r^2^ = 0.99648 for RAGE KO). There was no significant difference in the EC_50_ of CAP-evoked currents in WT (1.50 ± 1.29 µM) and RAGE KO (2.01 ± 1.04 µM; compared by unpaired *t* test). The concentration of 1 µM CAP was the submaximal working concentration used throughout the study.

### LPS potentiated CAP-evoked currents in tDRG neurons from WT, but not RAGE KO mice

**Figure 2:**
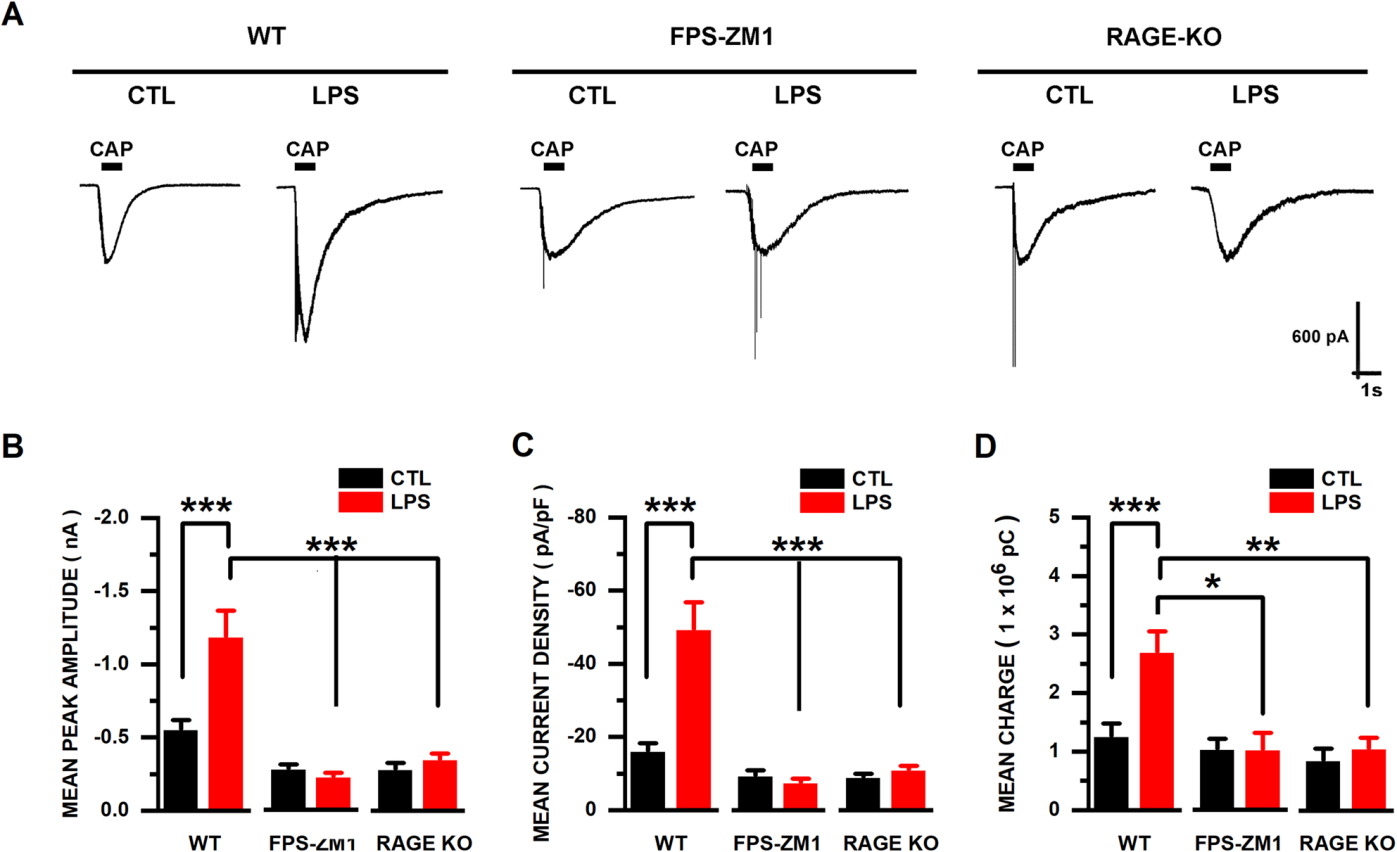
LPS exposure increases CAP-evoked currents in tDRG neurons from WT mice. (**A**) Representative CAP (1 µM; 1 s)-evoked current traces (V_H_ = − 60 mV) from cultured wild type (WT), neurons from WT mice treated in vitro with RAGE antagonist (10 μm; FPS-ZM1), and RAGE-knockout (RAGE KO) neonatal mouse tDRGs. (**B**) Mean peak amplitude (pA), (**C**) current density (pA/pF) and (**D**) charge (1 × 10^6^ pC) graphs of WT, FPS-ZM1, and RAGE KO tDRG neurons exposed to either control or LPS-containing media (1 µg/ml; 24 h). Treatment groups: WT control (n = 13); WT LPS (n = 9); FPS-ZM1 control (n = 6); FPS-ZM1 LPS (n = 5); RAGE KO control (n = 8); RAGE KO LPS (n = 9). Data represented as mean ± SEM. Means were statistically compared by 2-way ANOVA, followed by Tukey’s multiple comparison test; **p < 0.01; ***p < 0.001; ****p < 0.0001.

We recorded CAP-evoked currents in cultured tDRG neurons from WT mice maintained in control (CTL) conditions (n = 13) or incubated in LPS-containing growth media (for 24 h; n = 9). CAP-evoked currents in the LPS group had significantly higher mean peak amplitudes (− 1188.54 ± 177.76 pA) than controls (− 554.039 ± 64.01 pA; p < 0.0001, 2-way ANOVA and Tukey’s test; Fig. 2A,B). Similar results were found by calculating the current density (CTL − 16.19 ± 2.16 vs. LPS − 49.49 ± 7.38 pA/pF; p < 0.0001, 2-way ANOVA and Tukey’s test Fig. 2C) and ionic charge (CTL 1.26 ± 0.22 vs. LPS 2.70 ± 0.35 × 10^6^ pC/pF; p < 0.001, 2-way ANOVA and Tukey’s test Fig. 2D).

However, when tDRG neurons from WT mice were incubated (48 h) with the RAGE antagonist FPS-ZM1 (10 μM) together with LP (24 h), CAP-evoked currents maintained in control conditions (n = 6) or incubated in LPS (n = 5) did not exhibit significant differences. The mean peak amplitude (CTL − 271.16 ± 28.50 vs. LPS − 218.23 ± 26.68 pA; p = 0.9996; 2-way ANOVA and Tukey’s test), current density (CTL − 9.44 ± 3.44 vs. LPS − 7.54 ± 2.50 pA/pF; p = 0.9997; 2-way ANOVA and Tukey’s test), or ionic charge (CTL 1.03 ± 0.70 vs. LPS 1.04 ± 0.39 × 10^6^ pC; p = 0.9999; 2-way ANOVA and Tukey’s test) were not significantly different in the LPS group compared to controls.

Further confirmation of our results with the RAGE antagonist were obtained in tDRG neurons from RAGE-KO mice. CAP-evoked currents in neurons maintained in control conditions (n = 8) or incubated in LPS (n = 9) did not exhibit significant differences. The mean peak amplitude (CTL − 282.12 ± 43.79 vs. LPS − 349.77 ± 41.71 pA; p = 0.9954; 2-way ANOVA and Tukey’s test), current density (CTL − 9.00 ± 0.88 vs. LPS − 10.94 ± 1.14 pA/pF; p = 0.9989; 2-way ANOVA and Tukey’s test), or ionic charge (CTL 0.86 ± 0.20 vs. LPS 1.07 ± 0.18 × 10^6^ pC; p = 0.9924; 2-way ANOVA and Tukey’s test) were not significantly different in the LPS group compared to controls. Our results indicate that the lack of RAGE expression prevents the effects of LPS on CAP-evoked currents.

### LPS failed to increase neuronal excitability in tDRG neurons from RAGE KO mice

**Figure 3:**
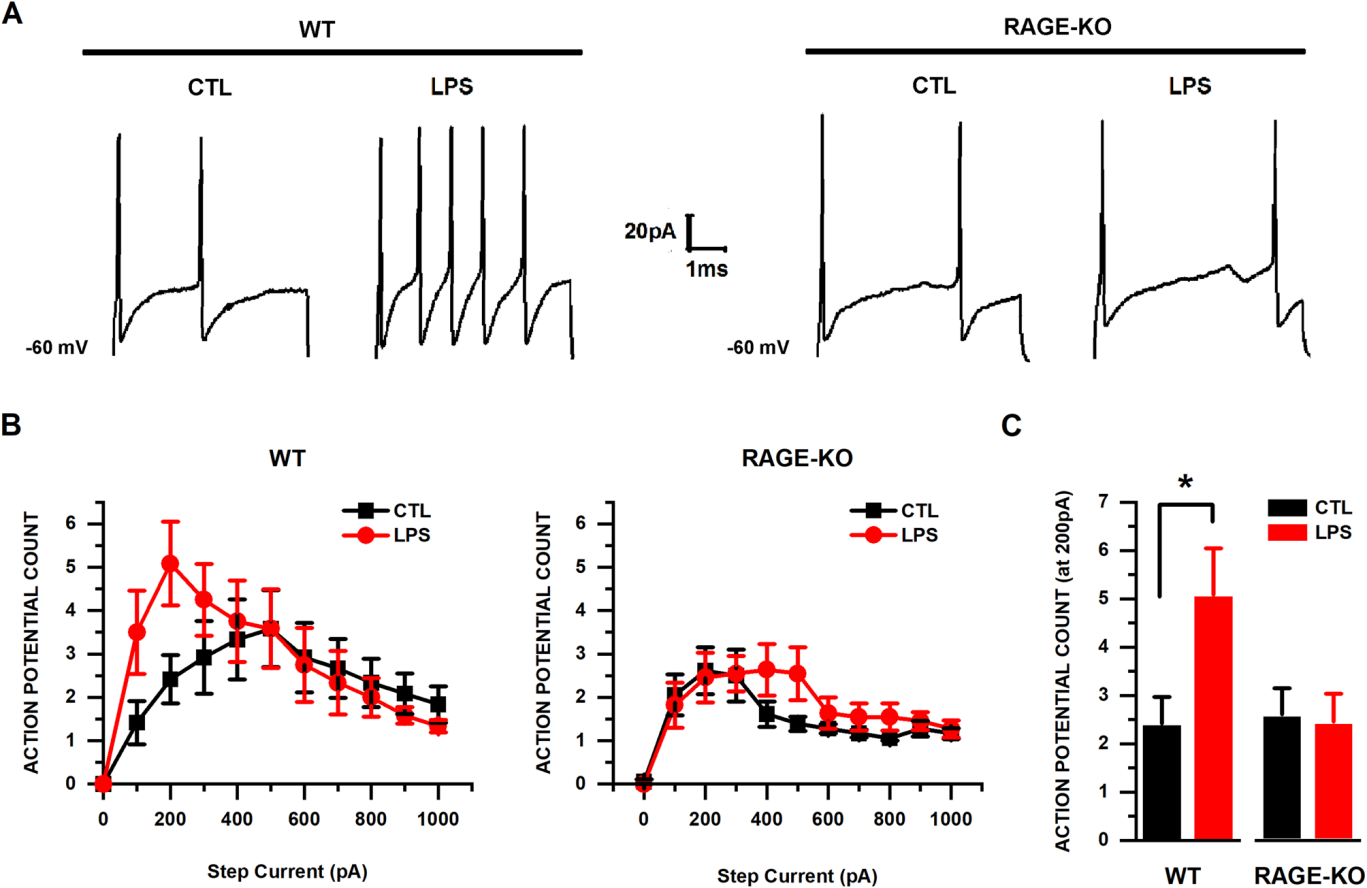
LPS exposure increases cell excitability in tDRG neurons from WT mice, but not from RAGE KO mice. (**A**) Representative action potential traces in response to the injection of a depolarizing current step (200 pA) in tDRG. (**B**) Mean action potential counts from cultured neurons from both genotypes. (**C**) Mean action potential count at the 200 pA current injection step. Treatment groups: WT control (n = 12); WT LPS (n = 12); RAGE KO control (n = 16); RAGE KO LPS (n = 11). Data presented as mean ± SEM. Means were statistically compared by 2-way ANOVA, followed by Tukey’s multiple comparison test; *p < 0.05.

To test whether the LPS-induced excitability of sensory neurons required RAGE expression, we evoked action potentials in cultured neurons from both WT and RAGE KO mice (Fig. 3A–C). We quantified action potentials generated by injection of depolarizing current steps (0–1000 pA, at 100 pA increments). Cultured tDRG neurons from WT mice treated with LPS displayed a significant increase in mean action potential counts at 200 pA (5.08 ± 0.96 counts; n = 12), compared to controls (2.42 ± 0.56 counts; n = 12; p = 0.0402; 2-way ANOVA and Tukey’s test; Fig. 3C). In contrast, in RAGE KO tDRG neurons there were no significant differences in action potential counts between the control (2.63 ± 0.60 counts; n = 16) and LPS groups (2.45 ± 0.58 counts; n = 11; p > 0.05; 2-way ANOVA and Tukey’s test). Also, there were no significant differences between resting membrane potential between tDRG neurons from WT and RAGE KO (Table 1; p > 0.05; 2-way ANOVA and Tukey’s test).

**Table 1:** Mean resting potentials.

	Experimental group			
	WT CTRL	WT LPS	RAGE KO CTRL	RAGE KO LPS
Mean VRM ± SEM (mV)	− 44.65 ± 2.43	− 48.20 ± 2.32	− 47.28 ± 1.56	− 47.66 ± 1.88

Combined mean resting membrane potential of WT and RAGE KO experimental groups recorded by whole-cell patch-clamp electrophysiology. Treatment groups: WT CTL (25); WT LPS (21); RAGE-KO CTL (24), and RAGE-KO LPS (20). Data represented as mean ± SEM. Means were statistically compared by 2-way ANOVA, followed by Tukey’s multiple comparison test.

### RAGE and neuronal activity are required for LPS-induced liquid secretion in mouse trachea

**Figure 4:**
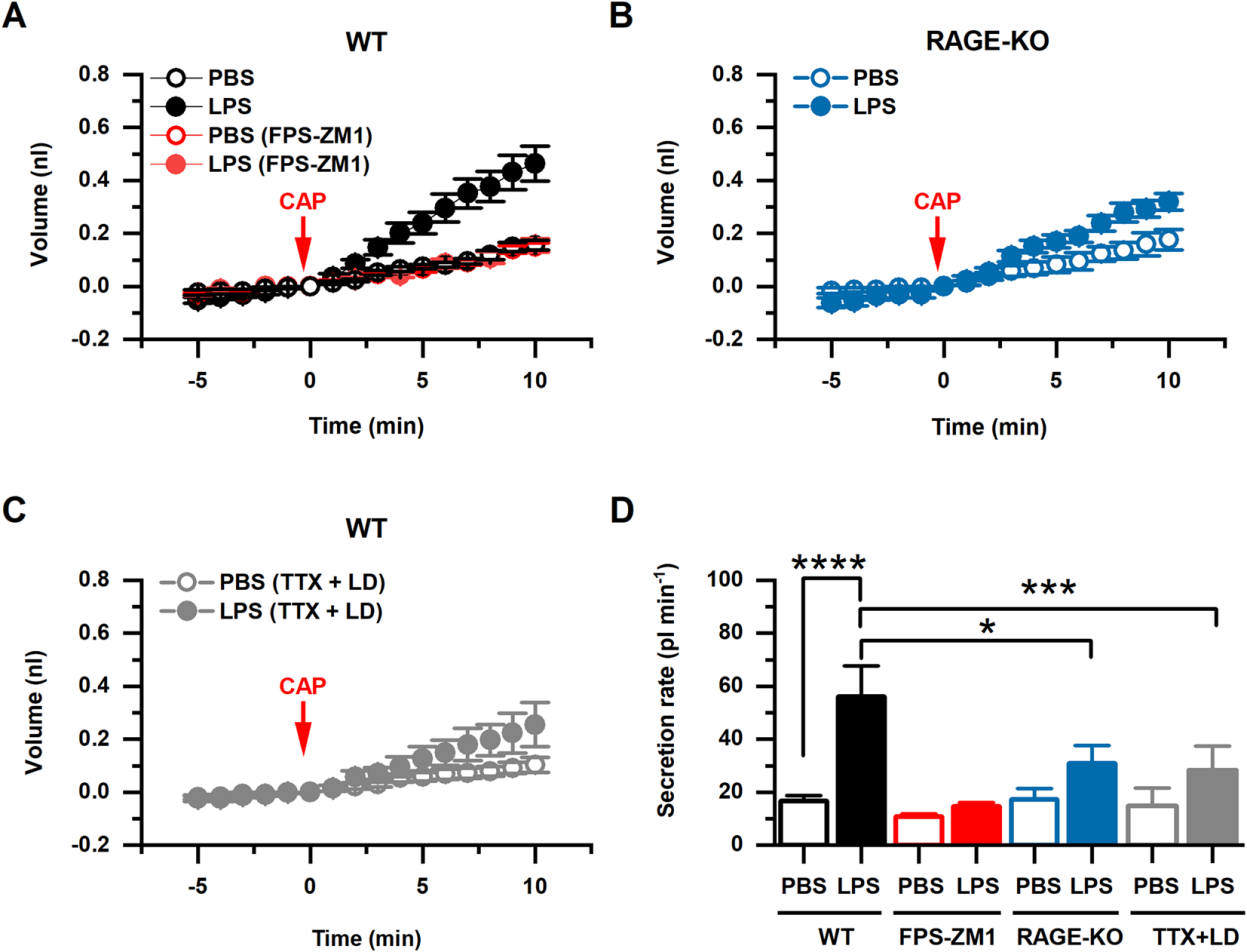
In vivo LPS instillation increased fluid secretion by tracheal submucosal glands, which was prevented by the absence of RAGE or by blocking neuronal activity. (**A**) Capsaicin (CAP) stimulated fluid secretion by tracheas dissected from WT mice treated with PBS (n = 6), LPS (n = 8), PBS (FPS-ZM1) (n = 5), and LPS (LPS-ZM1) (n = 5). (**B**) CAP stimulated fluid secretion by tracheas dissected from RAGE KO mice treated with PBS (n = 5) or LPS (n = 8). (**C**) CAP stimulated fluid secretion by tracheas bathed in vitro with the nervous system blockers TTX and lidocaine (TTX + LD), from animals treated in vivo with PBS (n = 7) or LPS (n = 7). (**D**) CAP stimulated fluid secretion rate (pl/min) in tracheas dissected from WT mice (PBS n = 11, LPS n = 17), WT mice treated with FPS-ZM1 (PBS n = 5, LPS n = 5), RAGE KO mice (PBS n = 5, LPS n = 8), and tracheas (from WT mice) treated in vitro with TTX + LD (PBS n = 7, LPS n = 7). Data presented as mean ± SEM. Means were statistically compared by 2-way ANOVA, followed by Tukey’s multiple comparison test; *, p < 0.05; ***, p < 0.001; **** p < 0.0001.

Treatment of WT mice with LPS in vivo induced an increased basal submucosal gland secretion (i.e., without stimulation, Fig. 4A) with a rate of 7.3 ± 2.5 (n = 6) and 19.6 ± 4.3 (n = 8) pl/min for PBS and LPS treated mice, respectively (p < 0.05, 2-way ANOVA and Tukey’s test, data not shown). Moreover, tracheas from WT mice treated with LPS triggered a significantly increased response to CAP, relative to control mice treated with PBS (Fig. 4A,D). CAP-stimulated secretion rate obtained from animals that were treated with PBS (control) was 15.60 ± 2.0 pl/min (n = 11), while in animals treated with LPS it was 51.89 ± 5.4 pl/min (n = 17, p < 0.0001; 2-way ANOVA and Tukey’s test), suggesting that exposure to bacteria induces an increased basal secretion and predisposes the airway to an increased response to CAP. In contrast, when RAGE-KO mice or WT mice injected with the RAGE antagonist FPS-ZM1 were treated with LPS, the response to CAP of the isolated trachea in the secretion assay preparation was not significantly different from those of animals treated with PBS. Secretion rates quantified after CAP stimulation in animals treated with FPS-ZM1 (48 h), were of 8.96 ± 2.04 pl/min in the PBS group versus 14.72 ± 1.35 pl/min (n = 5) in the LPS group (n = 5) (Fig. 4A,D, p = 0.9982; 2-way ANOVA and Tukey’s test). Furthermore, when the secretion assays were carried in tracheas form RAGE-KO mice we observed rates of 17.39 ± 3.89 pl/min in the PBS group pl/min (n = 5) versus 32.38 ± 3.22 in the LPS group (n = 8) (Fig. 4B,D, p = 0.6058; 2-way ANOVA and Tukey’s test). Thus, the ability of the airway to hyper secret in response to LPS exposure is abolished in the absence of RAGE expression.

To test whether this heightened response to CAP was mediated by the nervous system, we repeated the experiments using TTX and lidocaine to block neuronal function. When tracheas isolated from WT mice treated with either PBS or LPS were incubated with 1 µM TTX and 100 µM lidocaine (TTX + LD) before exposure to CAP, the basal secretion rates were not significantly different from preparations not exposed to these neuronal blockers (p > 0.05, data not shown). The tracheas from LPS-treated mice incubated in TTX + LD failed to display the increased CAP-induced secretory response. When tracheas were incubated in TTX + LD, the secretion rates after stimulation with CAP were not significantly different from control (without TTX + LD); 11.88 ± 2.23 pl/min (n = 7) and 22.66 ± 7.24 pl/min (n = 7) for PBS and LPS groups respectively (Fig. 4C,D, p = 0.7118; 2-way ANOVA and Tukey’s test). These results suggest that the LPS-induced pro-inflammatory state triggers hypersecretion of airway liquid by the submucosa glands via the RAGE-dependent sensitization of the nervous system.

### LPS induces changes in the protein expression profile of tDRG neurons

To determine whether LPS increased RAGE expression in tDRG neurons, we used two approaches; first, the detection of RAGE in whole tDRGs from mice treated intranasally with LPS compared to those treated with PBS; and second, the global protein analysis in tDRG neurons from WT mice exposed in culture conditions to either control or LPS.

**Figure 5:**
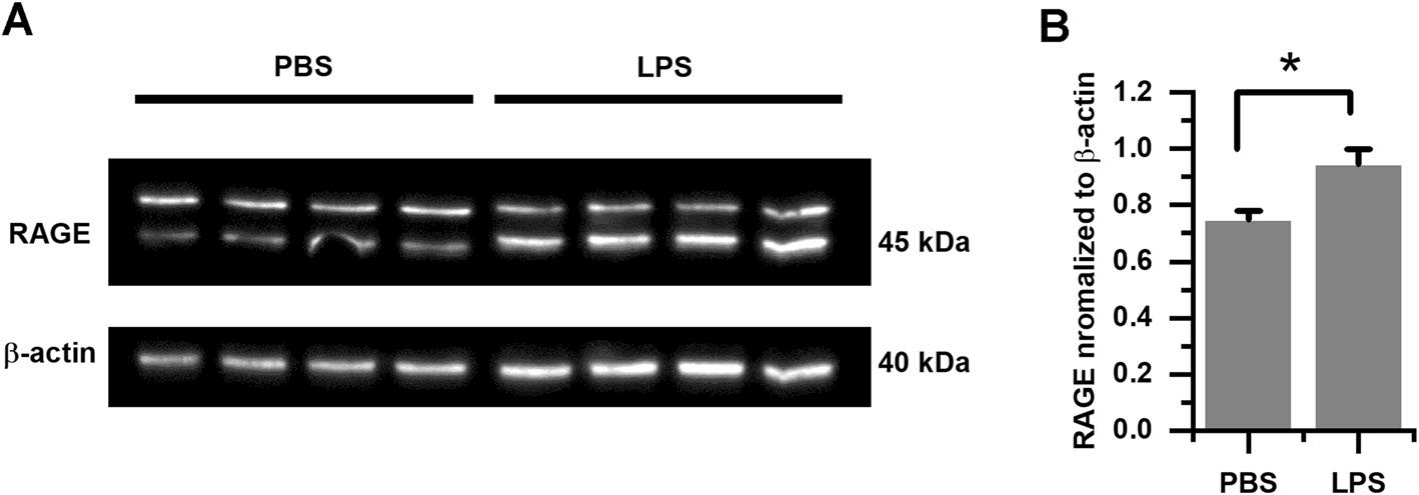
In vivo LPS instillation increased RAGE expression in WT mice. (**A**) Immunoblot showing levels of RAGE expression, and the loading control β-actin, in whole tDRGs from LPS-treated mice compared to PBS-treated controls. (**B**) Bar graphs show the mean ± SEM levels of RAGE protein after normalization to β-actin. Data represented as mean ± SEM. Means were statistically compared by Mann–Whitney test. *p < 0.05.

We observed a significant difference in the expression levels of RAGE in whole tDRGs from WT mice treated intranasally with LPS compared to animals treated with PBS. First, we collected tDRGs from WT mice treated intranasally with LPS and detected RAGE expression levels by Western blotting (Fig. 5; p = 0.0286; Mann–Whitney test; full blots provided in “Supplementary data”). Next, using mass spectrometry (MS)-based proteomics analysis, we detected global protein changes in neuron-enriched samples from cultured tDRGs (Table 2). We report 65 proteins that showed a change in the total protein spectral intensities by at least twofold difference between LPS-treated and control tDRG cultures. For full-length RAGE, encoded by Tv1-RAGE (acc. #: AAH61182.1^35^), there was a 4.1 fold change increase in total intensity difference. We also detected the sRAGE splice variant, encoded by *Mmus*RAGEv4 (acc. #: ADX07280^35^), which showed a 0.3 fold change decrease.

**Table 2:** Global protein changes detected by mass-based spectrometry analysis.

Accession	Protein	FC	Protein regulation^a^
**RAGEs**			
AAH61182.1	RAGE (Tv1-RAGE, full length)	4.1	Up
ADX07280	RAGE specific variant 4 (*Mmus*RAGEv4)	0.3	Down
**KINASES**			
Phosphoinositide 3-kinases (PIk3) and regulatory proteins			
CAB89686	Phosphoinositide 3-Kinase gamma (PI3K γ)	6.6	Up
AAN05615	Phosphoinositide 3 kinase P110delta (PI3K δ)	2.6	Up
NP_001075035	Phosphoinositide 3-kinase regulatory subunit 6 isoform 1 (PIK3-6)	13.6	Up
XP_006527535	Phosphoinositide 3-kinase adapter protein 1 (PIK3ap1)	2.2	Up
NP_033782	AKT1 kinase	3.4	Up
**PKCs**			
NP_035234	Protein kinase C epsilon type (PKC-Ɛ)	2.0	Up
NP_035233	Protein kinase C delta type (PKC-δ)	2.8	Up
NP_032882	Protein kinase C eta type (PKC-ƞ)	3.6	Up
AAI27084	Protein kinase C beta type (PKC-β)	2.4	Up
NP_035231	Protein kinase C alpha type (PKC-α)	3.0	Up
NP_032885	Protein kinase C theta type (PKC-Ɵ)	2.1	Up
NP_032883	Protein kinase C iota type (PKC-Ɩ)	0.4	Down
**MAPK/ERKs**			
NP_001344044	Mitogen-activated protein kinase 1 (MAPK 1)	2.1	Up
NP_036082	Mitogen-activated protein kinase 3 (MAPK 3)	2.4	Up
AAH14830	Dual specificity mitogen-activated protein kinase kinase 2 (MAP2K2)	2.8	Up
**MAPK/Jnks**			
Q61831	MAPK 10; Alt Name: c-Jun N-terminal kinase 3 (JNK3)	2.7	Up
Q9WTU6	MAP kinase 9; Alt Name: c-Jun N-terminal kinase 2 (JNK2)	3.3	Up
Q91Y86	MAP kinase 8; Alt Name: c-Jun N-terminal kinase 1 (JNK1)	2.2	Up
**MAPK/P38K**			
NP_038899	MAPK 12; Alt Name: MAP kinase p38 gamma (p38MAPK- γ)	4.1	Up
Q9Z1B7	MAPK 13; Alt Name: MAP kinase p38 delta (p38MAPK-δ)	3.5	Up
Q9WUI1	MAP kinase p38 beta, partial (p38MAP-κ)	8.7	Up
**Src kinase family**			
AAX90616	Src	2.8	Up
**NADPH**			
NP_001272762	NADPH oxidase 4	2.2	Up
**Phosphatases**			
NP_032986	Phosphatidylinositol 3,4,5-trisphosphate 3-phosphatase and dual-specificity protein phosphatase PTEN	0.4	Down
**cAMP related proteins**			
AAL47131.1	cAMP response element binding protein (CREB)	2.1	Up
AAL90757	cAMP response element binding protein 1 (CREB1)	2.2E + 07	Up
**Calcium signaling**			
AAH30071	Calcitonin/calcitonin-related polypeptide (CALC)	2.3	Up
NP_079669	Protein S100-A14 isoform a	10.0	Up
EDL15111	S100 calcium binding protein A8 (calgranulin A)	25.1	Up
AAH86903.1	S100 calcium binding protein A11 (calgizzarin)	5.5E + 06	Up
EDL15122	S100 calcium binding protein A16, isoform CRA_a	3.7	Up
EDL31858	S100 protein, beta polypeptide, neural, isoform CRA_a	2.0E + 07	Up
XP_017174998	S100-A3 isoform X1	3.7	Up
AAH05590	S100 calcium binding protein A1	9.9E + 05	Up
XP_006501694	S100-A15A isoform X1	3.8	Up
XP_011238348	S100-A13 isoform X1	2.5	Up
EDL15114	S100 calcium binding protein A6 (calcyclin)	3.4	Up
EDL15110	S100 calcium binding protein A9 (calgranulin B), isoform CRA_a	2.5	Up
EDL15116	S100 calcium binding protein A4	6.8	Up
AAH10751	S100 calcium binding protein G	1.2E − 07	Down
AAI47384	S100 calcium binding protein, zeta	2.0E − 07	Down
**NFkB and associated factors**			
NP_032715	Nuclear factor kappa-B p105 subunit; Alt name P50 (NFkB p105)	8.1	Up
NP_001170840	NFkB p100 subunit isoform a	6.5E + 05	Up
XP_006540014	NFkB inhibitor delta isoform X2 (NFkBID)	8.5E − 09	Down
NP_001293151	NFkB inhibitor beta (NFkBIB)	0.4	Down
**TNF and associated factors**			
EDL12915	Tumor necrosis factor (TNF) receptor associated factor 4, isoform CRA_c	2.1	Up
XP_006533219	PREDICTED: TNF receptor-associated factor 4 isoform X1 (Traf-4)	2.6	Up
EDL12971	TNF receptor-associated factor 5 (Traf-5)	2.0	Up
NP_001265530.1	TNF isoform 2 (TNF-2)	7.9E + 06	Up
NP_001313530	TNF receptor-associated factor 1 (Traf1)	10.2	Up
EDL27657	TNF receptor-associated factor 6, isoform CRA_a (Traf6)	0.4	Down
**Interleukins and IL-associated kinases**			
EDL28238	interleukin (IL)-1 β (IL-1β)	2.9	Up
NP_001300983	IL-6 isoform 2 precursor (IL-6)	0.4	Down
EDL00308	IL-8 receptor, α (IL-8Rα)	316.9	Up
EDL39722	IL-10	2.8	Up
NP_001171447.1	IL-1 receptor-associated kinase 1 isoform 2	4	Up
NP_001171444	IL-1 receptor-associated kinase 1 isoform 1 (IRAK1)	6.0E + 07	Up
NP_084202	IL-1 receptor-associated kinase 4 (IRAK4)	3.2	Up
CAD29448	IL-1 receptor-associated kinase M	0.5	Down
NP_001171318	Toll/IL-1 receptor domain-containing adapter protein	0.1	Down
**Other proteins**			
A2ASI5	Sodium channel protein type 3 subunit alpha (Nav1.3 α)	2.4	Up
Q8BYH8	Chromodomain-helicase-DNA-binding protein 9 (CHD-9); Alt Name: PPAR-α-interacting complex protein	2.0	Up

Fold change determined from total protein spectral intensity of RAGE isoforms and downstream signaling pathways from cultured tDRG neurons maintained in either control conditions or incubated in LPS for 24 h. To classify proteins as up- or down-regulated, we considered the fold change (FC) of ≥ 2 and < 0.5 in spectral intensities with respect to control.

^a^Up-regulated proteins—FC ≥ 2; Down-regulated proteins—FC < 0.5.

In addition, exposure of tDRG neurons to LPS altered the expression profile of nicotinamide adenine dinucleotide phosphate oxidase (NADPH oxidase), for which LPS caused a 2.2 fold change increase (Table 2). This was consistent with the reported activation of RAGE through NADPH oxidase in sensory neurons leading to the formation of ROS, a known RAGE-mediated effect in diabetic neuropathy^50,51^.

We also detected changes in the two main signalling pathways downstream from RAGE, the Mitogen-Activated Protein Kinases (MAPK) and the phosphoinositide 3-kinase (PI3K) pathways^52^. LPS treatment induced changes in MAPK/ERK, including increases in MAPK 1, MAPK 3, and dual-specificity mitogen-activated protein kinase kinase 2 (MAPK2K), of 2.1, 2.4, and 2.8-fold change increases, respectively (Table 2). In the MAPK/c-Jun N-terminal kinase (JUNK) pathway, JNK 1, JNK 2 and JNK 3, showed 2.2, 3.3, and 2.7 fold change increases respectively, and in the MAPK/P38 mitogen-activated protein kinases (P38) pathway, P38MAK-δ, P38MAPK-γ, and P38MAPK-κ showed 3.5, 4.1, and 8.7 fold change increases, respectively (Table 2). In the PI3K/AKT pathway, we detected a 3.4 fold change increase in AKT1, consistent with recruiting the pro-survival pathway (Table 2). In addition, the phosphatase and tensin homolog (PTEN), which negatively regulates the PI3K/Akt pathway was decreased by a 0.4 fold change (Table 2).

Furthermore, LPS induced increases in other RAGE-downstream kinases, such as a 2.8 fold change increase in the proto-oncogene tyrosine-protein kinase Src (Src)^53,54^, and various PKC isoforms including PKC-α, -β, -δ, -ε, -η, and -θ from 2.0 to 3.6 fold change increases. In contrast, PKC-Ɩ, was decreased by a 0.4 fold change (Table 2).

Exposure of tDRG neurons to LPS also triggered activation of pro-inflammatory signalling proteins, including multiple variants of S100 calcium-binding proteins, particularly S100A1, S100A11, and S100β, which showed 9.9E + 05, 5.5E + 06, and 2.0E + 07 fold change increases, respectively (Table 2). We also detected a marked increased (6.5E + 05 fold change) in the pro-inflammatory nuclear factor kappa B (NFkB), while the NFkB inhibitor δ showed a marked 8.5E-09 fold change decrease (Table 2). Consistent with increased RAGE-NFkB activation^55,56^, we observed a dramatic increase in the levels of cAMP response element binding protein 1 (CREB1) of 2.2E + 07 fold change increase (Table 2). A number of interleukins (ILs), including IL1, IL6, IL8, and IL10 were found to be increased, in particular, IL8 with a 316.9 fold change increase (Table 2). In addition, the interleukin-1 receptor-associated kinase 1 (IRAK1) showed a marked 6.0E + 07 fold change increase by LPS treatment, and we also detected increases in various members of the tumor necrosis factor superfamily, in particular isoform 2 (TNF-2) which showed a 7.9E + 06 fold change increase (Table 2).

Lastly, we detected a 2.4 fold change increase of the sodium channel protein Nav1.3 α-subunit, which is consistent with increased neuronal excitability in tDRG neurons treated with LPS (Table 2). In contrast, a 2.0 fold change increase of peroxisome proliferator activator receptor-α (PPAR-α) (Table 2) suggests activation of homeostatic mechanism against the pro-inflammatory effects of LPS^57,58^.

## Discussion

### LPS-induced sensitization of tDRG neurons

We studied sensitization in sensory neurons from DRGs corresponding with the thoracic spinal region (T1–T4) because they provide sensory innervation to the upper airways^6,7^. Sensory afferent neurons from tDRG and the jugular/nodose ganglia co-innervate submucosal glands in the upper airways^1,2,43,59^. As reported in this study, and by others^60–63^, exposure of DRG neurons to LPS induced electrophysiological phenomena consistent with neuronal sensitization, such as the potentiation of CAP-evoked currents and the increase in action potential generation. We also report a new piece of information linking LPS with these electrophysiological parameters of sensitization—we found that the potentiation effect triggered by LPS required functional expression of RAGE.

The effect of bacterial LPS on sensory neurons has been extensively used as a tool to study inflammatory signaling^60–63^. LPS binds to TLR4, resulting in the induction and release of pro-inflammatory cytokines from macrophages and sensory neurons^64^. However, LPS can also physically interact with RAGE^14,65^, triggering a RAGE-mediated regulation of inflammatory responses in a mouse model of septic shock^14^. For instance, some effect of LPS seem to be mediated solely by RAGE, such as the induction of acute lung injury and acute respiratory distress syndrome^66^. However, the high mobility group box 1 (HMGB1) protein, which is an endogenous ligand for TLR4 and RAGE, can cause differential degrees of activation of each receptor depending on the red-ox state. The latter results in two possible functional isoforms, an all-thiol HMGB1 (at-HMGB1) that activates RAGE^55^, or a disulfide HMGB1 (ds-HMGB1) that activates TLR4^67,68^. Furthermore, it has been reported that TLR4 activation by LPS, requires RAGE co-activation^69^. We found that LPS induced sensitization in tDRG neurons from WT mice but failed to do so in neurons from RAGE-KO mice or from WT treated in vitro with the RAGE antagonist FPS-ZM1.

Furthermore, LPS failed to exacerbate glandular responses to CAP in tracheas from RAGE KO mice or from animals that were treated with the RAGE antagonist FPS-ZM1. Thus, our findings are consistent with the activation of the RAGE pathway by LPS; however, we cannot eliminate a possible direct or indirect contribution from an LPS-mediated activation of TLR4 in our observations.

We use the response of cultured tDRG neurons to CAP as an assay to study sensory physiology. We chose to study TRPV1 because of its role in initiating protective reflexes such as cough and mucus secretion in response to irritants^5,70^. Most of the current knowledge on the link between LPS and TRPV1 functions comes from research in pain signaling. It was previously reported that when in sensory neurons TLR4 (which usually co-localizes with TRPV1) is activated by LPS, it results in increased TRPV1 expression, which in turn increases pain signaling^60,71^.

Confirmation of the relationship between LPS and RAGE in tDRG neurons was obtained by Western blotting and mass spectrometry. Our proteomics data helps visualize the landscape of changes that take place in tDRGs when they are exposed to LPS. For instance, LPS induced a 1.6 fold change increase in TRPV1 expression; however, it did not reach the criteria of at least > twofold change (thus not included in Table 2). This suggests that the increased CAP-evoked response observed in our electrophysiological studies does not seem to be completely explained by increased TRPV1 expression alone. The potentiation of TRPV1 responses after LPS treatment could also be mediated by posttranslational modifications, independent of expression levels. As previously reported by us^23^ and others^72–74^, cytosolic ROS potentiate CAP-evoked currents. Chuang et al.^74^ demonstrated that ROS mediate the modification of cysteine residues within the TRPV1 channel protein. The interaction of LPS with its receptors indeed leads to the production of cytosolic ROS, particularly to generate hydroxyl radicals^75^, which could, therefore, potentiate TRPV1 responses.

### LPS responses in the trachea

Our findings revealed that instilling LPS into the airway of mice triggered a hypersecretory responses to stimulants. We can draw two major conclusions from this part of the study; first, that the effect of LPS on the CAP-induced secretory response in the trachea of mice required the expression and function of RAGE; and second, that the effect of LPS was mediated through the nervous system. Capsaicin acts on TRPV1 receptors expressed on sensory neurons that innervate submucosal glands directly, or indirectly by modulating the airway intrinsic plexus^2^. Studies in the rat trachea, show that TRPV1 was expressed primarily in sensory neurons innervating intra- and sub-epithelial parts of the trachea, in the form of isolated fibers or dense networks. In addition, TRPV1 co-localized with the peptidergic neuron marker substance P and calcitonin gene-related peptide (CGRP)^76^. Confirmation of the source of this sensory innervation was provided by retrograde labelling from the trachea, which shows that cell bodies of labeled fibers concentrated in the DRG and jugular/nodose ganglia. In contrast, airway intrinsic neurons from the peritracheal plexus did not express TRPV1, and they showed marked expression of the heat-sensitive TRPV2 receptor, which was not co-localized with peptidergic marker^76^.

Thus, the tDRG neurons we studied in the current report, together with those in the jugular/nodose ganglia, are responsible for the CAP-evoked responses in the trachea. The fact that we can stimulate liquid secretion in isolated trachea preparations, which lack tDRG and jugular/nodose ganglia, indicates that the sensory fibers remain viable/active for at least 60 min (the duration of the experiment) after euthanizing the animal and that the effects of LPS on CAP-mediated secretory responses are the result of local mechanisms, such as axon reflex. The latter is consistent with previous reports by Ianowski and coworkers, indicating that nerve conduction blockers impaired secretory responses in trachea isolated from mice^43^ and swine^77^. Because sensory neurons from the tDRG and jugular/nodose ganglia are virtually identical regarding responses to CAP, we cannot discard the contribution of vagal afferents to the secretory responses obtained in our study. We can, however, correlate the effects caused by LPS on tDRG neurons in culture with those of LPS on the trachea secretion essays. Therefore, our data strongly support the idea that tDRG sensory neurons (and likely those in jugular/nodose ganglia) are sensitized by LPS through a RAGE-mediated pathway, causing increased neuronal excitability and increased liquid secretion in the upper airways.

### Insights from proteomic profiling of tDRG samples

Our proteomics study revealed a wide scope of the cytosolic changes that correlate with the electrophysiological responses observed in tDRGs after LPS treatment. We report here that the LPS-induced changes observed in cultured tDRG were concomitant with the upregulation of full-length RAGE, encoded by Tv1-RAGE, and downregulation of an sRAGE splice variant, encoded by *Mmus*RAGEv4.

So far, 17 splice variants of RAGE have been described^35^, from which only 3, in addition to full-length RAGE, contain coding sequences for a transmembrane domain and cytosolic tail, and therefore, have the capacity to activate the signalling cascade. The splice variant encoded by *Mmus*RAGEv4 lacks exons 8 and 9 (transmembrane domain) but contains the three Ig domains and the signal peptide. This particular variant has been previously identified in embryonic mouse tissues as a coding version of RAGE; although, no physiological function has been associated with it so far. Due to the lack of a transmembrane domain in the variant encoded by *Mmus*RAGEv4, it has been proposed as a soluble RAGE (or sRAGE) splice variant^35^. Soluble versions of RAGE can be generated by different mechanisms. Removal of the transmembrane domain allows the generation of sRAGE isoforms by different biological processes, either by alternative splicing (esRAGE) or proteolytic cleavage^78^ of carboxyl-terminal truncation of membrane-bound isoforms (cRAGE)^79^ by the sheddase ADAM10^80^. The sRAGE isoforms act as decoys for AGE and other RAGE ligands and are considered cytoprotective^81^. However, the complex equilibrium of membrane-bound RAGE, sRAGE, esRAGE, and RAGE ligands remain largely unclear. It has been previously reported that a direct correlation exists between sRAGE and risk of both diabetic and cardiovascular complications^82,83^. However, in a different population, decreased concentrations of sRAGE have been linked with increased risk of complications^84^. Furthermore, Kang et al.^85^, have described an sRAGE variant in pancreatic cancer cells with cytosolic functions, which upon pERK1/2-mediated phosphorylation, translocates to the mitochondria and stimulates ATP biosynthesis required for tumor growth.

While we can only speculate that the variant encoded by *Mmus*RAGEv4 detected in tDRG neurons is indeed part of the sRAGE pool, its downregulation under our experimental LPS-induced conditions seems consistent with a reduced protective effect of sRAGE variants as described in some neurodegenerative diseases^86^. The lack of a transmembrane domain could render a protein to be localized either extracellularly or in the cytoplasm. Further research is required to address this point, in particular, determining whether the variant encoded by *Mmus*RAGEv4 in tDRGs has cytosolic functions such as those described by Kang et al.^85^.

### Proposed working model for the LPS-mediated sensitization of tDRG neurons

**Figure 6:**
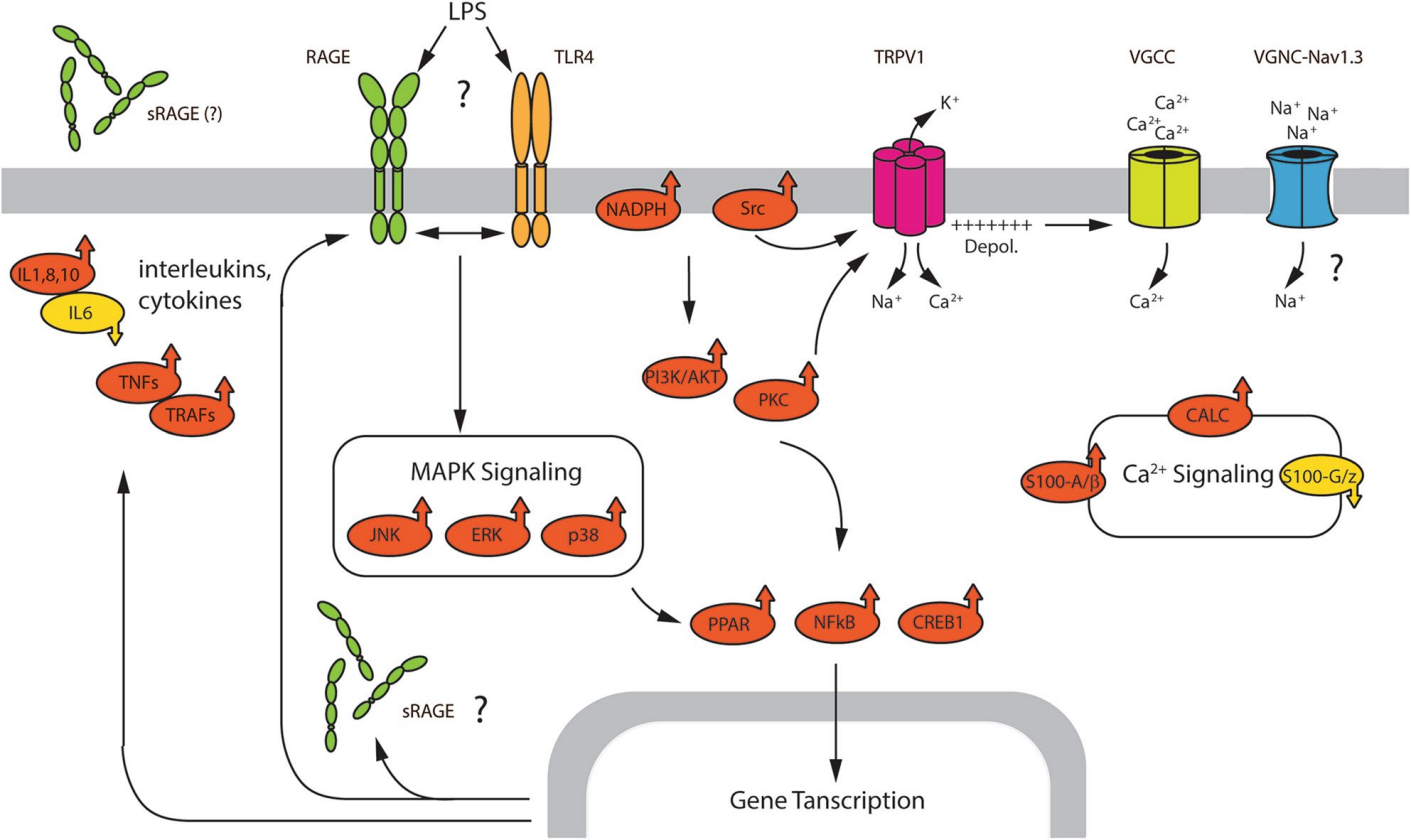
Working model of LPS-induced RAGE signaling in sensory neurons innervating the upper airways. The schematic diagram summarizes the global protein changes in intracellular pathways and membrane receptors detected by in tDRG neurons from WT mice exposed to either PBS (control) or LPS, as revealed by mass spectrometry (MS)-based proteomics. Pathway proteins in red-arrow up indicate protein upregulation, and in yellow-arrow down indicate downregulation. All abbreviations correspond to nomenclature used in Table 2, except for: VGCC, voltage-gated Ca^2+^ channels; VGNC-Nav1.3, voltage-gated Na^+^ channel containing the Nav1.3 subunit.

The schematic diagram in Fig. 6, shows a potential working model for the biochemical changes that LPS induce in tDRG based on correlating our findings obtained from cellular electrophysiology, trachea fluid secretion, and global tDRG proteomics. LPS interacts with RAGE, possibly in combination with TLR4, leading to the activation of membrane-bound NADPH oxidase and the subsequent production of cytosolic ROS. LPS also increased the expression of multiple PKC isoforms, including PCKβ and Src kinases. PKCβII and Src have been reported to phosphorylate TRPV1 channels and thus increasing its activity^87,88^. Furthermore, TRPV1 function can also be increased by ROS-mediated cysteine modification^74^ due to the activation of NADPH oxidase. LPS increased expression levels of the α subunit of voltage-gated sodium channel type 3 (Nav1.3) in our tDRG samples. Although the involvement of Nav1.3 in sensory physiology remains controversial, it has been reported that its absence, by Nav1.3 antisense construct or Nav1.3 null mice, reduced the hyperexcitability of dorsal horn neurons and attenuated pain-related behaviors associated with chronic constriction injury of the peripheral nerve^89,90^.

We also detected increased expression levels of multiple members of the S100 protein family, including S100A9, which is elevated in the sputum of asthmatic patients^91^, and S100A1 and S100A11, which are considered markers of airway inflammatory conditions such as COPD^92,93^. We also detected marked changes in other S100 members of the family, particularly S100G and S100Z, which showed decreased expression. However, the role of these isoforms in airway inflammation or sensory function is not yet known.

While the LPS-mediated upregulation of ROS, Src, S100 proteins, and Nav1.3 could underlie the sensitization of tDRG neurons, the upregulation of proteins such as PPAR-α could minimize it. PPAR-α has been shown to beneficially regulate LPS-induced inflammation in many mammalian cells, including fibroblasts^57^ and lung epithelial cells^58^. Therefore, further research is needed to investigate whether PPAR-α may play a neuroprotective role, such as described for other members of the protein family (i.e. PPAR-ɣ in amyotrophic lateral sclerosis (ALS)^94^). Furthermore, it has been recently reported that PARP-1 regulated TNF-α expression in the DRG and spinal dorsal horn contributing to the development and maintenance of neuropathic pain in mice^95^.

Changes in TNF isoforms, together with interleukins and interleukin associated kinases further depicts the inflammatory environment triggered by LPS in tDRG neurons. TNF has been linked to upper airway inflammation; we detected a marked increase in TNF2, which has been proposed as a marker in bronchitis^96,97^. The increase in cytosolic ROS downstream from RAGE activation, perhaps linked to NADPH oxidase upregulation, is a key initiator of the PI3K/AKT and MAPKs signalling pathways. Interestingly, we have detected a decrease in PTEN, which is known to suppress LPS-induced lung fibroblast proliferation and differentiation through inhibition of the PI3K/AKT/GSK3β pathway^98^. Thus, our data of increased PI3K-associated kinases is consistent with the decreased PTEN expression after LPS treatment in tDRG.

With respect to MAPK signalling, we detected changes in the three pathways, ERK, JNK, and p38. We detected a number of kinases associated to three MAPK pathways (Table 2) that showed upregulation, demonstrating strong recruitment of MAPK-dependent signalling in response to LPS treatment. Therefore, our findings are consistent with previous reports, by others and us, showing increased signalling through the MAPKs pathway upon RAGE activation^25–30,38,99^. We also detected changes in the transcription factors CREB1 and NFkB. CREB is induced by growth factors and inflammatory signals, and in turn, it mediates the transcription of genes containing a cAMP-responsive element^100^. Multiple interleukins and members of the TNF family contain this element, which is consistent with our findings. The link between inflammatory mediators (e.g., interleukins and TNF), CREB activation, and airway secretion has been established before^46,101^. Also, phosphorylated CREB has been proposed to directly inhibit NF-κB activation by blocking the binding of CREB binding protein to the NF-κB complex, thereby limiting pro-inflammatory responses. We detected a marked increase in NFkB p100 subunit and marked decrease in NFkB inhibitor δ isoform. The latter is consistent with the activation of the positive feedback upon RAGE activation that leads to further RAGE expression^102^, including alternative splicing generating sRAGE variant encoded by *Mmus*RAGEv4 (possibly extracellular or cytoplasmic, indicated by a question mark) in addition to the full-length membrane RAGE, encoded by Tv1-RAGE.

## Supplementary Information

Supplementary Information.
