# Supplementary material for: Lipopolysaccharides induce a RAGE-mediated sensitization of sensory neurons and fluid hypersecretion in the upper airways
Source: Sci Rep. 2021 Apr 16;11:8336. doi: 10.1038/s41598-021-86069-6 (PMC8052339; doi:10.1038/s41598-021-86069-6)

Lipopolysaccharides induce a RAGE-mediated potentiation of sensory neurons and fluid hypersecretion in the mouse trachea

Manoj Nair, Santosh Jagadeeshan, George Katselis, Xiaojie Luan, Zeinab Momeni, Nicolas Henao-Romero, Paulos Chumala, Julian S. Tam, Yasuhiko Yamamoto, Juan Ianowski, Verónica A. Campanucci

MS-based proteomic analyses - Supplementary Methods

1. MS-based proteomics analyses. Suspensions of cultured tDRG neurons from C57BL/6 WT and RAGE KO mice were prepared as described in the main text, and plated onto specialized cell culture dishes (Sarstedt, Nümbrecht, Germany). We used approximately 80,000 cells per experimental group and each plating was generated from 20 pups. Established DRG cultures were incubated for 24h at 37°C; (5% CO2; humidified) in growth media alone (control) or with 1µg/ml LPS (Sigma-Aldrich, MO, USA)-supplemented growth media. Membrane and soluble fractions from cultured tDRG neurons were obtained using the ProteoExtract Native Membrane Protein Extraction Kit (EMD Millipore, MA, USA) and processed as per the manufacturer's instructions and stored at -80°C. Protein concentrations were determined by Nanodrop analysis with a BioTek ELx808 Synergy HY multi-detection plate reader (BioTek Instruments Inc., VT, USA).

1.1 In-solution Digestion. Protein samples were concentrated by acetone precipitation. To precipitate proteins, 4x sample volume of cold (-80^{0}C) acetone was added to an aliquot from lysate, vortexed, and incubated overnight at -80^{0}C. Then samples were centrifuged 18000 x *g* at 4^{0}C for 15 min. After washing protein pellets twice with cold (-80^{0}C) 80% acetone/20% water, protein pellets were air dried and re-suspended in 55 µl trifluoroethanol (TFE; Fisher Scientific, Fair

Lawn, NJ, USA) buffer (10% TFE, 100 mM ammonium bicarbonate (ABC)). Proteins were digested in-solution using an in-house developed protocol. Briefly, 45 µl of each protein sample was placed in a 1.5 ml tube and diluted with 5 µl of 1 M ABC buffer (Fisher Scientific, Fair Lawn, NJ, USA) and 50 µl TFE to denature proteins. The samples were treated with 1 µl of 1M DTT (dithiothreitol) (MP Biomedicals, Solon, OH, USA) while shaking at 300 RPM (Eppendorf Thermomixer, Eppendorf, Mississauga, ON, Canada) at 60°C for 60 min to reduce disulfide bonds. Next, samples were alkylated with 100 µl of 110 mM iodoacetamide (IAA; Fisher Scientific, Fair Lawn, NJ, USA) at 37°C for 30 min on a shaker, covered with aluminum foil, to prevent further disulfide bond formation. The samples were dried in a speedvac (Labconco, Kansas City, MO, USA). Proteins in the samples were treated with 1 ml cold acetone followed by refrigeration at -80°C for 60 min to eliminate salts and other interfering compounds (e.g. detergents), which can prevent digestion. The samples were centrifuged twice at 18000 x *g* for 30 min and acetone was carefully removed. Next, samples were dried in a speedvac and a buffer-containing trypsin (Promega Corporation, Madison, WI, USA) solution (50 ng/µl trypsin in 1 mM HCl (hydrochloric acid) /100 mM ABC) was added to the samples in a 40:1 protein:trypsin ratio. The samples were incubated in a shaker at 300 RPM overnight at 37°C. Trypsin buffer at the same ratio was added again in the morning to ensure complete digestion of proteins into peptides. After 2 hrs of further incubation at 37°C, digested peptides were dried in speedvac and stored at -80°C until further analysis.

1.2 Strong Cation Exchange (SCX)-based Fractionation. SCX-based fractionation was performed using SCX SpinTips sample prep kit (Protea Biosciences, Morgantown, WV, USA). The digested protein samples were dissolved in 200 µl of SCX reconstitution solution (pH 3). The

samples were then loaded on to SCX SpinTip and centrifuged at 2000 x *g* for 6 min. To enhance peptide binding to SCX SpinTip samples were reloaded and centrifuged 3 more times. The flow through at the end of the step was transferred to a 1.5 ml tube for MS analysis. Peptides bound to SCX column were eluted using stepwise concentrations (in M): 20, 40, 60, 80, 100, 150, 250, 500 of ammonium formate (Sigma-Aldrich) in 10% acetonitrile (ACN; Fisher Scientific) at pH 3. 150 µl of ammonium formate (in increasing concentrations) was added to the SpinTip and centrifuged at 2000 x *g* for 6 min. The flow through was collected after every centrifugation and transferred to 1.5 ml tube. All flow throughs were dried in speedvac and stored at -80°C.

1.3 MS Workflow. All SCX fractions containing tryptic peptide were reconstituted in 20 µl of MS grade water:ACN:formic acid (FA) (97:3:0.1 v/v) followed by vortexing for 1-2 min. The resulting solutions were centrifuged at 18000 x *g* for 10 min at 4ºC. 15 µl aliquot of each sample was transferred to a mass spectrometry vial (Agilent Technologies Canada Ltd., Mississauga, ON, CA) for liquid chromatography-tandem mass spectrometry (LC-MS/MS) analysis. All MS analyses were performed on an Agilent 6550 iFunnel quadrupole time-of-flight (QTOF) mass spectrometer equipped with an Agilent 1260 series liquid chromatography instrument and a Chip Cube LC-MS (liquid chromatography-mass spectrometry) interface (Agilent Technologies). Chromatographic peptide separation was accomplished using a high-capacity high performance liquid chromatography (HPLC)-Chip II: G4240-62030 Polaris-HR-Chip_3C18 consisting of a 360 nl enrichment column and a 75 µm × 150 mm analytical column, which were both packed with Polaris C18-A, 180Å, 3 µm stationary phase. Samples were loaded onto the enrichment column with 50% solvent A (0.1% FA in water) and 50% solvent B (0.1% ACN:FA) at a flow rate of 2.0 µl/min. Samples loaded onto the enrichment column were transferred onto analytical column, and

peptides were separated with linear gradient solvent system. The linear gradient program was employed for peptide separation with solvent A and solvent B on an analytical column. The linear gradient was 3–25% solvent B for 50 min and then 25–90% solvent B for 10 min at a flow rate of 0.3 µl/min. Positive-ion electrospray MS data were acquired using a capillary voltage set at 1900 V, the ion fragmentor set at 360 V, and the drying/collision gas (nitrogen) set at 225 ºC with a flow rate of 12.0 l/min. Spectral results were collected over a mass range of 250–1700 mass/charge (m/z) at a scan rate of 8 spectra/sec. Tandem mass spectrometry (MS/MS) data were collected over a range of 100–1700 m/z and a set isolation width of 1.3 atomic mass units. The top 20 most intense precursor ions for each MS scan were selected for MS/MS with active exclusion for 0.25 min.

1.4 Protein Identification. Tandem mass spectra were extracted from raw data and were processed against the NCBI (National Center for Biotechnology Information) non-redundant *Mus musculus* database as well as custom database (containing all known mouse RAGE protein isoforms), using Spectrum Mill (Agilent Technologies) as the database search engine. Search parameters included a fragment mass error of 50 parts per million (ppm), a parent mass error of 20 ppm, trypsin cleavage specificity, and carbamidomethyl as a fixed modification of cysteine. In addition, four stages of database search in variable modification mode were carried out with different sets of variable modifications. In the first stage, carbamylated lysine, oxidized methionine, pyroglutamic acid, deamidated asparagine and phosphorylated serine, threonine, and tyrosine were set as variable modifications. In the second stage, validated hits from the first stage were searched using the following variable modifications: acetyl lysine, oxidized methionine, pyroglutamic acid, deamidated asparagine, and phosphorylated serine, threonine, and tyrosine.

The validated hits from the second stage were searched using semi-trypsin non-specific C-terminus, yielding the third stage validated hits, which were subsequently searched using semi-trypsin non-specific N-terminus (fourth stage), with no other variable modifications specified. After each stage, Spectrum Mill validation was performed at peptide and protein levels (1% false discovery rate, FDR), and spectral counts and intensities were used to report relative quantitation of proteins. The Mass Profiler Professional (MPP, version 15.0, Agilent, Santa Clara, CA, USA) software was used for statistical analysis using one-way ANOVA. A cut-off value of $p < 0.05$ and the Benjamini and Hochberg FDR set at < 1% were used to obtain statistically significant results. In addition, a fold change (FC) of ≥ 2 and < 0.5 in spectral intensities with respect to control were considered to classify proteins as up- and down-regulated, respectively, in LPS samples.

Supplementary information for Figure 5: Full blot images.

Western Blotting - RAGE

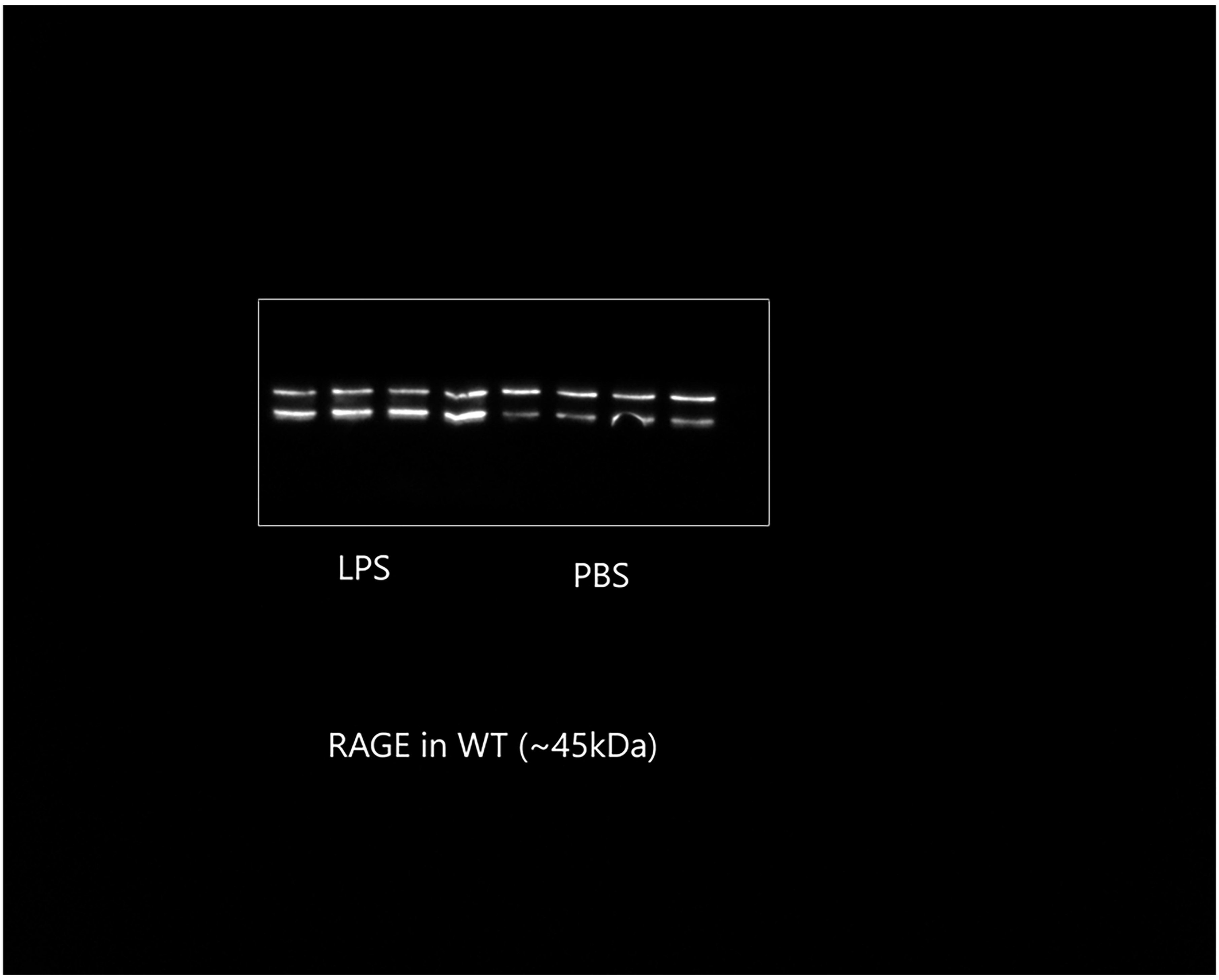

Western blotting - β-actin

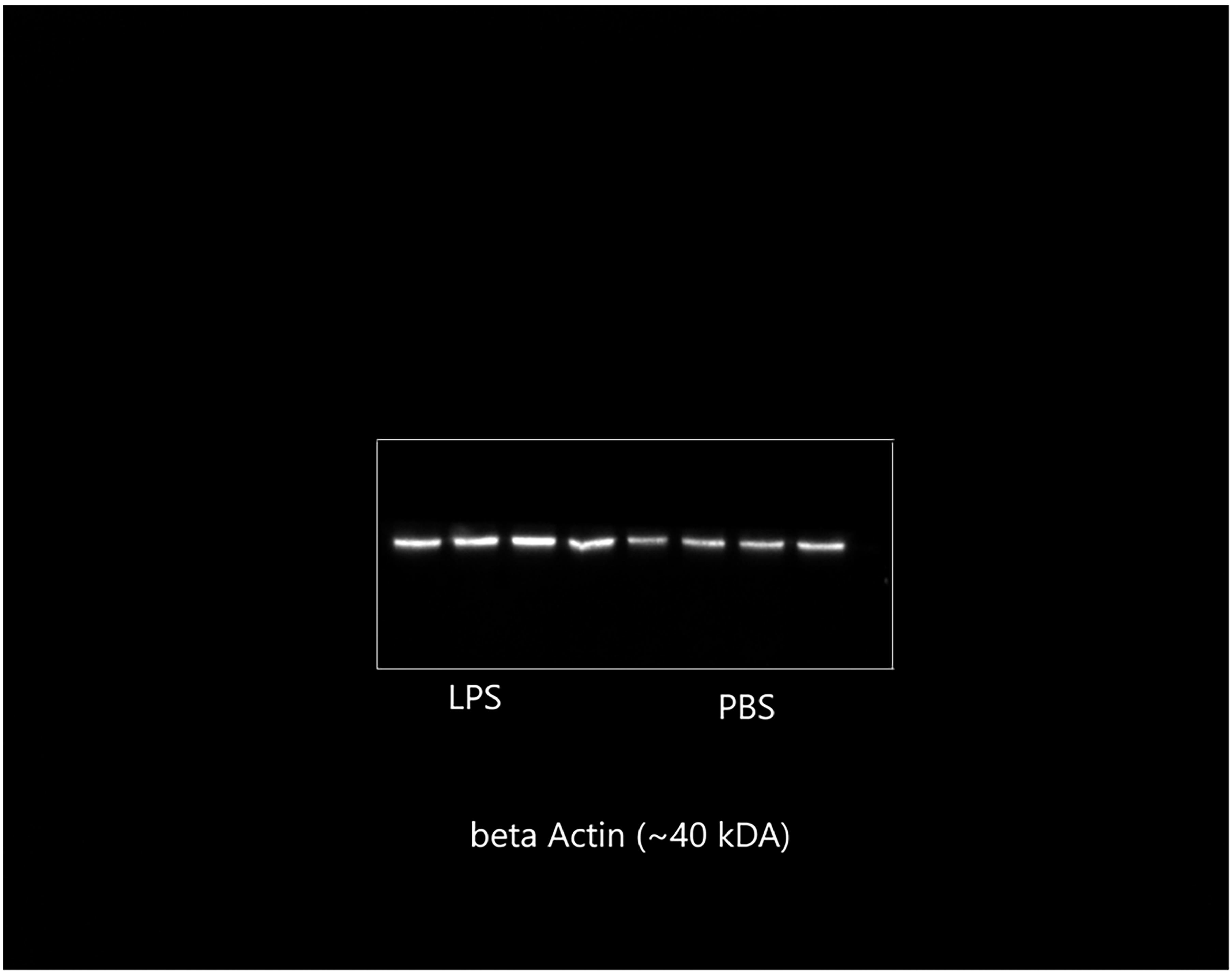

Supplement: Supplementary file 1 — Supplementary Information. [file 41598_2021_86069_MOESM1_ESM.pdf]
